# The Influence of Sex on Clinical Outcomes after Surgical Mitral Valve Replacement in Spain (2001–2015)

**DOI:** 10.3390/jcm9124108

**Published:** 2020-12-19

**Authors:** Nuria Muñoz-Rivas, Ana López-de-Andrés, Manuel Méndez-Bailón, Emmanuel Andrès, Valentín Hernández-Barrera, José María de Miguel-Yanes, Javier de Miguel-Díez, Noel Lorenzo-Villalba, Rodrigo Jiménez-García

**Affiliations:** 1 Internal Medicine Department, Hospital Universitario Infanta Leonor, 28031 Madrid, Spain; nmrivas@hotmail.com; 2 Department of Public Health & Maternal and Child Health, Faculty of Medicine, Universidad Complutense de Madrid, 28040 Madrid, Spain; ana.lopez@urjc.es (A.L.-d.-A.); valentin.hernandez@urjc.es (V.H.-B.); rodrijim@ucm.es (R.J.-G.); 3 Internal Medicine Department, Hospital Universitario Clínico San Carlos, Universidad Complutense de Madrid, 28040 Madrid, Spain; manuel.mendez@salud.madrid.org; 4 Service de Médecine Interne, Diabète et Maladies Métaboliques, Hôpitaux Universitaires de Strasbourg, 67000 Strasbourg, France; emmanuel.andres@chru-strasbourg.fr; 5 Internal Medicine Department, Hospital General Gregorio Marañon, 28007 Madrid, Spain; josemaria.demiguel@salud.madrid.org; 6 Respiratory Department, Hospital General Universitario Gregorio Marañón, 28007 Madrid, Spain; javier.miguel@salud.madrid.org

**Keywords:** sex, hospital admissions, mitral valve replacement surgery, in-hospital mortality

## Abstract

(1) Background: Mitral regurgitation (MR) is the second most prevalent valvular heart disease in developed countries. Mitral valve (MV) disease is a common cause of heart failure and a leading cause of morbidity and mortality in the U.S.A. and Europe. (2) Methods: We performed a retrospective study using the Spanish National Hospital Discharge Database, 2001–2015. We included patients that had surgical mitral valve replacement (SMVR) listed as a procedure in their discharge report. We sought to (i) examine trends in incidence of SMVR among women and men in Spain, (ii) compare in-hospital outcomes for mechanical and bioprosthetic SMVR by sex, and (iii) identify factors associated with in-hospital mortality (IHM) after SMVR. (3) Results: We identified 44,340 hospitalizations for SMVR (84% mechanical, 16% bioprosthetic). The incidence of SMVR was higher in women (IRR 1.51; 95% CI 1.48–1.54). The use of mechanical SMVR decreased over time in both sexes and the use of bioprosthetic valves increased over time in both sexes. Men who underwent mechanical and bioprosthetic SMVR had higher comorbidity than women. IHM was significantly lower in women who underwent SMVR than in men (10% vs. 12% *p* < 0.001 for mechanical and 14% vs. 16% *p* = 0.025 for bioprosthetic valve, respectively). Major adverse cardiovascular and cerebrovascular events (MACCE) were also significantly lower in women who underwent mechanical and bioprosthetic SMVR. A significant reduction in both in-hospital MACCEs and IHM was observed over the study period regardless of sex. After multivariable logistic regression, male sex was associated with increased IHM only in bioprosthetic SMVR (OR 1.28; 95% CI 1.1–1.5). (4) Conclusions: This nationwide analysis over 15 years of sex-specific outcomes after SMVR showed that incidences are significantly higher in women than men for mechanical and bioprosthetic SMVR. IHM and MACCE have improved over time for SMVR in both sexes. Male sex was independently associated with higher mortality after bioprosthetic SMVR.

## 1. Introduction

Mitral regurgitation (MR) is the second most prevalent valvular heart disease in developed countries [1]. Mitral valve (MV) disease is a common cause of heart failure and is one of the leading causes of morbidity and mortality in the U.S.A. and Europe [2]. The prevalence is approximately 10% in people older than 75 years [3]. MR is classified into primary MR, most commonly degenerative, and secondary (functional) MV regurgitation, in which ischemic or non-ischemic dilatation of the left ventricle or mitral annulus leads to abnormal geometry with consequent insufficiency [4]. These entities represent different disease processes, have different treatment approaches, and also different prognoses. Mitral valve repair (MVR) or prosthetic replacement surgery are the treatments of choice for MR. Valve repair for primary MR represents the gold standard with established high-quality results in reference centers [5], whereas in functional regurgitation, MVR is associated with high failure rates with progressive ventricular dysfunction There are cases in which the mitral valve is not repairable, and replacement is a safer option. In those cases, the replacement can be performed with a mechanical or a bioprosthetic valve. The choice of prostheses has not been given appropriate attention within the literature. Jamieson et al. [6] concluded that porcine bioprosthesis is satisfactory for implantation in patients older than 70 years of age but not in younger patients. 

There has been a resurgence in interest for surgical mitral valve replacement (SMVR), especially in the management of functional MR [7]. The results of a randomized controlled trial comparing MV repair and chordal-sparing MV replacement in patients with severe ischemic MR showed a higher incidence of recurrent MR one year after MV repair with no difference in survival [8]. Thus, current guidelines of American College of Cardiology/American Heart Association task force give a IIa level of recommendation for chordal-sparing MV replacement versus MV repair in this patient cohort [9,10].

The influence of sex on SMVR outcomes is still unclear. Studies conducted in the U.S.A. and Germany assessed the impact of female sex on clinical outcomes following mitral valve surgery and concluded that there are substantial differences regarding MV pathology, surgical strategy, and long–term outcomes [11]. Chiu Wong et al. [12] found that women after isolated left-sided heart valve surgery experienced higher in-hospital major adverse cardiac and cerebral events (MACCE) including all-cause mortality compared to men. Previously, Vassileva CM et al. [13], reported a higher operative mortality and lower long-term survival following mitral valve surgery in women using the Center for Medicare and Medicaid Services data from 2000 to 2009. With the aging population in the coming years, particularly in the proportion of female sex, the incidence of MR is likely to continue to increase. Knowledge regarding sex-specific MR outcomes is lacking in Spain. It is important to understand the sex-specific differences in treatment approaches and clinical outcomes following mitral valve surgeries. 

The aim of the present study was to examine nationwide trends in SMVR from 2001 to 2015 in Spain using the Spanish National Hospital Discharge Database (SNHDD). We assessed longitudinal trends, clinical characteristics, and in-hospital outcomes among women and men according to implanted valve type (mechanical or bioprosthetic). Additionally, we identified factors associated with in-hospital mortality (IHM) among women and men according to the implanted valve type.

## 2. Materials and Methods

### 2.1. Data Source

The SNHDD covers more than 95% of hospital admissions in Spain and contains nationwide information of up to 14 discharge diagnoses and up to 20 procedures performed during the hospital stay obtained from the discharge report [14]. ICD-9-CM is used for coding in the SNHDD. For this study, data from 2001 to 2015 were used.

### 2.2. Study Population

We selected admissions of adult patients (aged ≥ 18 years) whose medical procedures included SMVR (ICD-9-CM codes: 35.23 and 35.24) in the SNHDD database.

### 2.3. Covariates

Clinical characteristics included an assessment of overall comorbidity at the time of discharge, which was calculated using the Charlson comorbidity index (CCI) [15]. The clinical diagnosis and procedures analyzed in our investigation and the corresponding ICD-9-CM codes are shown in Supplementary Table S1. 

Among the procedures we specifically analyzed, we selected records for patients in whom coronary artery bypass graft (CABG), surgical aortic valve replacement (SAVR), surgical procedures on pulmonary and/or tricuspid valves, intra-aortic balloon counterpulsation, pacemaker implantation, and blood transfusions were performed during the hospitalization for the mitral valve replacement. To assess the etiology of mitral regurgitation, we identified records containing the code 394.1 for rheumatic mitral insufficiency in any diagnosis field.

We evaluated the mean of length of hospital stay (LOHS) and in-hospital mortality (IHM) as the proportion of patients who died during admission for each year of the study, then estimated the incidence of peri-operative MACCE. As described by Newman et al. [16], MACCE includes acute myocardial infarction, ischemic stroke, or death during the hospitalization.

### 2.4. End Points

The main end points in our investigation were trends in incidence rates of hospitalizations and IHM stratified according to the type of valve used (mechanical and bioprosthetic) in the SMVR for women and men separately.

### 2.5. Statistical Analysis

To describe the characteristics of the study samples, we used five time-periods that included three consecutive years each (2001–2003; 2004–2006; 2007–2009; 2010–2012; 2013–2015). In order to assess temporal trends, we estimated the yearly incidence rates of admission for mitral valve replacement in women and men per 100,000 inhabitants, applying the methods described in previous studies [17], and used multivariable Poisson regression analysis adjusted by age. 

A descriptive statistical analysis was performed for all continuous and categorical variables. Variables are expressed as proportions and as means with standard deviations. A bivariable analysis according to time period for clinical variables was performed using the χ^2^ test for linear trend and ANOVA, as appropriate.

To identify variables associated with IHM, we performed multivariable logistic regression analyses, one for each type of mitral valve replacement (mechanical and bioprosthetic) and for each sex and both sexes. The variables included in the multivariable models were those with significant results in the bivariable analysis and those considered relevant in other investigations. Estimates were expressed as odds ratios (ORs) with their 95% CI.

In order to control the confounding effect of covariates and to assess the effect of sex on IHM after SMVR, we conducted propensity score matching (PSM) models using logistic regression to obtain matched groups of women and men for comparative outcomes analysis. PSM was performed for bioprosthetic and mechanical SMVR patients separately. The variables included in the model were year of surgery, age, concomitant interventions, and comorbid conditions shown in Supplementary Table S2. 

All statistical analyses were performed with Stata version 10.1 (Stata, College Station, TX, USA). Statistical significance was set at *p* < 0.05 (2-tailed).

## 3. Results

We identified a total of 44,340 hospitalizations of patients aged 18 years or more who underwent SMVR in Spain between 2001 and 2015. We identified 37,177 (84%) hospitalized patients who underwent mechanical, and 7163 (16%) who underwent bioprosthetic mitral valve replacement.

### 3.1. Temporal Trends in Mechanical Mitral Valve Replacement Hospitalizations

The proportion of patients receiving a mechanical valve decreased from 91% in 2001–2003 to 77% in 2013–2015 (*p* < 0.001). Women showed a decrease from 92% to 78% and men showed a decrease from 90% to 76% between 2001–2003 and 2013–2015, respectively (*p* < 0.001 in both cases).

**Figure 1:**
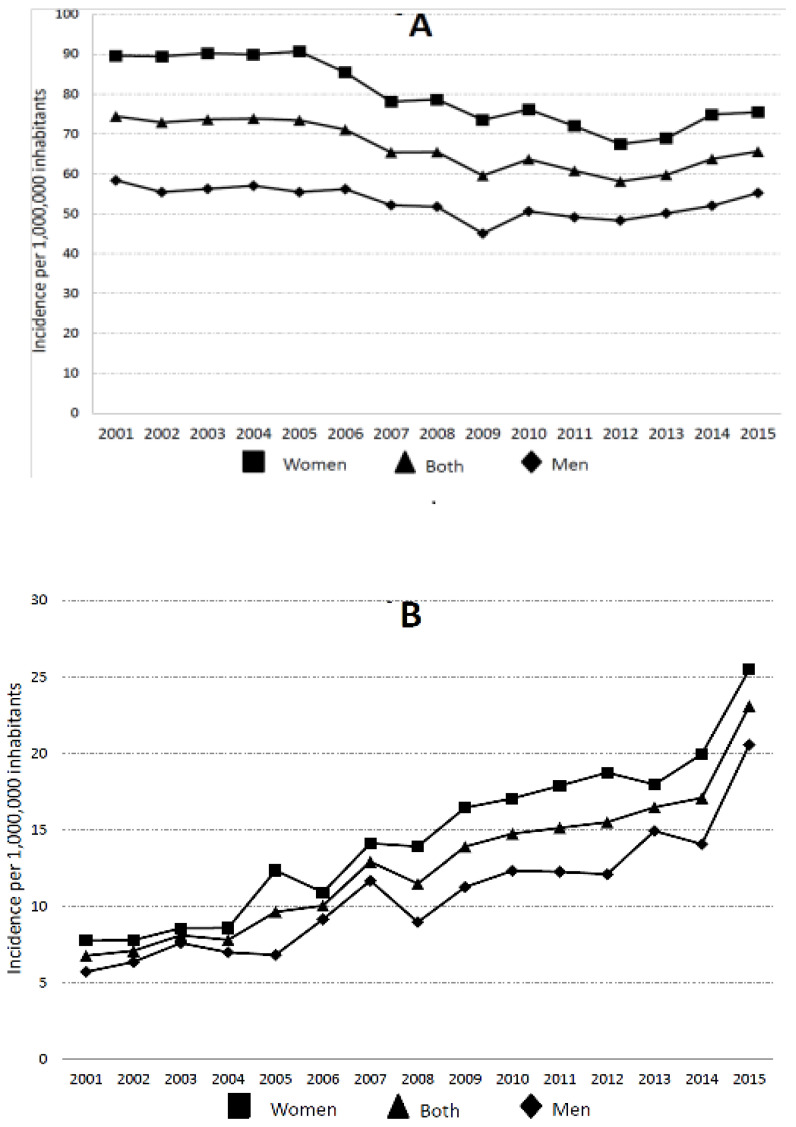
Trends in the hospitalization in patients undergoing mechanical (**A**) and bioprosthetic (**B**) mitral valve replacement according to sex (no. cases/1,000,000 inhabitants).

We found that the incidence of mechanical mitral valve replacement coding decreased significantly from 74 cases per 1,000,000 inhabitants in 2001 to 66 cases in 2015 (*p* < 0.001). Among women, the total incidence of mechanical valve replacement decreased from 90 cases in 2001 to 75 cases in 2015 (*p* < 0.001). In men, the incidence also decreased from 58 in 2001 to 55 in 2015 (*p* < 0.001). The incidence was significantly higher in women than in men for all years analyzed (Figure 1).

The Poisson regression models, conducted to assess the association of sex with the risk of admission for SMVR during the period 2001–2015, yielded an adjusted IRR for women who underwent mechanical valve replacement of 1.51 (95% CI 1.48–1.54).

Patient age increased significantly in both groups of patients (mean 61 years in men and 63 years in women in 2001–2003 vs. 64 and 65 years old, respectively, in 2013–2015; all *p* < 0.001). There was a significant increase in the frequency of SAVR, other valve procedures on pulmonary or tricuspid valves, pacemaker implantation, and blood transfusion in women and men over time (Supplementary Table S2). The use of concomitant CABG remained stable.

Mean LOHS for men and women undergoing mechanical mitral valve replacement was 24 and 22 days, respectively, in the period 2001–2003, decreasing to 21 and 19 days, respectively, in 2013–2015 (all *p* < 0.001). In men, IHM and MACCE decreased significantly over time from 14% and 19%, respectively, in 2001–2003 to 10% and 15% in 2013–2015 (all *p* < 0.001). Among women, IHM (11% to 7%) and MACCE (13% to 9%) decreased over time (all *p* < 0.001) (Supplementary Table S2). As can be seen in Supplementary Table S2, only around 4% of men and women who underwent a mechanical mitral valve replacement in Spain from 2001 to 2015 had a diagnosis code for rheumatic mitral insufficiency. This proportion remained stable over time and was similar in both sexes.

Compared with women, men were younger at the time of intervention (62 ± 11 vs. 64 ± 10; *p* < 0.001) and were more likely to undergo simultaneous CABG (17% vs. 7%), SAVR (34% vs. 31%, *p* < 0.001) and intra-aortic balloon counterpulsation (5% vs. 2%, *p* < 0.001). In contrast, women had nearly twice as many other valve procedures on the pulmonary or tricuspid valves compared to men (31% vs. 17%, *p* < 0.001) (Supplementary Table S2).

LOHS was higher in men than in women, and crude IHM and MACCE were significantly higher in men (12% and 17% vs. 10% and 12%) (Supplementary Table S2). 

We found a significant increase in comorbidity according to the mean CCI over time in both men and women (0.90 and 0.80, respectively, in 2001–2003 vs. 1.15 and 1.09, respectively, in 2013–2015; all *p* < 0.001). The most common associated comorbidities for hospitalized men who underwent mechanical mitral valve replacement were atrial fibrillation (49%), coronary artery disease (28%) and congestive heart failure (23%), while in women they were atrial fibrillation (63%), pulmonary hypertension (32%) and congestive heart failure (20%). In both groups the frequency of type 2 diabetes (T2DM), peripheral vascular disease, acute renal disease, congestive heart failure, atrial fibrillation, obesity, endocarditis, renal disease, liver disease, and weight loss increased significantly over time, while pneumonia showed a significant decrease in men and women over time (3% and 3%, respectively, to 2% and 2%). Cerebrovascular disease, coronary artery disease, cardiogenic shock, and cancer increased significantly over time only in men. In women, the frequency of pulmonary hypertension increased during the study period (28% vs. 34%) (Table 1).

Men had more comorbidity than women (mean CCI 1.03 (SD 0.98) vs. 0.95 (SD 0.92), *p* < 0.001) and had higher rates of chronic obstructive pulmonary disease (COPD), peripheral vascular disease, congestive heart failure, coronary artery disease, cardiogenic shock, endocarditis, pneumonia, renal disease, liver disease, and cancer. However, women had significantly higher frequency of T2DM (16% vs. 14%), cerebrovascular disease (5% vs. 5%), atrial fibrillation (63% vs. 49%), pulmonary hypertension (32% vs. 21%), and obesity (8% vs. 5%) compared to men who underwent mechanical mitral valve replacement (Table 2).

In both groups, mean LOHS, IHM, and MACCE decreased significantly over time. In men, IHM and MACCE were 19% and 26%, respectively, in the period 2001–2003, decreasing significantly to 14% and 18%, respectively, in 2013–2015. The same trends were seen in women (*p* < 0.001), with IHM falling from 18% to 14% and MACCE from 22% to 18% (Table 2).

In men, comorbidity according to the mean CCI increased over the study period from 1.11 ± 1.07 in 2001–2003 to 1.37 ± 1.05 in 2013–2015 (*p* < 0.001). In women, the mean CCI increased significantly from 0.93 ± 0.93 in the first period to 1.23 ± 1.03 in the last period. In both groups, the most common associated comorbidities for hospitalized patients who underwent bioprosthetic mitral valve replacement were the same as those described in patients who underwent mechanical mitral valve replacement. The prevalence of acute renal disease, congestive heart failure, atrial fibrillation, pulmonary hypertension, endocarditis, and renal disease increased significantly over time in both groups. In men, the frequency of peripheral vascular disease and obesity increased between 2001–2003 and 2013–2015. However, in women, the prevalence of gastrointestinal hemorrhage and pneumonia decreased from 2% and 5%, respectively, in 2001–2003 to 0% and 3% in 2013–2015 (Table 3).

Men had higher levels of comorbidity than women (mean CCI 1.26 ± 1.05 vs. 1.12 ± 0.99, *p* < 0.001) and had higher rates of peripheral vascular disease (7% vs. 3%), congestive heart failure (29% vs. 27%), coronary artery disease (37% vs. 22%), cardiogenic shock (6% vs. 4%), endocarditis (24% vs. 15%), pneumonia (5% vs. 3%), renal disease (12% vs. 8%), liver disease (5% vs. 3%), and cancer (2% vs. 1%). However, women had a significantly higher frequency of T2DM, atrial fibrillation, pulmonary hypertension and obesity compared to men who underwent mechanical mitral valve replacement (Table 3).

### 3.2. Differences between Patients Admitted for Mechanical versus Bioprosthetic Mitral Valve Replacement

When we compared patients who underwent mechanical SMVR with patients who underwent bioprosthetic SMVR, we found that the mechanical valve replacement male and female patients were younger than those who received bioprosthetic valves (62.5 and 64.3 years, respectively, vs. 72.1 and 73.4 years, respectively; *p* < 0.001). Men and women who received bioprosthetic versus mechanical valves had higher comorbidity and were more likely to require concomitant CABG, SAVR, other valve procedures on pulmonary or tricuspid valves, intra-aortic balloon counterpulsation, pacemaker implantation, and blood transfusion. In addition, they had longer LOHS, higher IHM (in men: 16% vs. 12% and in women: 14% vs. 10%), and higher values of MACCE (in men: 21% vs. 17% and in women: 18% vs. 12%) compared with those patients who received mechanical valves (all *p* < 0.001) as can be seen in Supplementary Table S2 and Table 2.

### 3.3. Factors Associated with IHM

The factors independently associated with IHM according to the type of mitral valve are shown in Table 4.

Regardless of the type of valve implanted and for both sexes, factors that increased IHM included older age, intra-aortic balloon counterpulsation during admission, acute renal disease, congestive heart failure, cardiogenic shock, endocarditis, pneumonia, and liver disease. 

Other valve procedures on pulmonary or tricuspid valves, blood transfusion during admission, cerebrovascular disease, coronary artery disease, gastrointestinal hemorrhage, and renal disease were associated with an increase in risk of IHM in men and women with mechanical mitral valve replacement. Among women, IHM was significantly higher in those with SAVR (OR 1.24; 95% CI 1.11–1.38). Men with cancer had a 1.6-fold higher risk of dying compared to men without this comorbidity (OR 1.6; 95% CI 1.06–2.42).

For women who underwent bioprosthetic mitral valve replacement, factors that increased IHM included blood transfusion during the admission (OR 1.66; 95% CI 1.34–2.05), peripheral vascular disease (OR 2.42; 95% CI 1.49–3.93), cerebrovascular disease (OR 1.83; 95% CI1.24–2.7), and renal disease (OR 1.97; 95% CI 1.45–2.69). Among men who underwent bioprosthetic valve, IHM was significantly higher in those with SAVR (OR 1.32; 95% CI1.05–1.67).

Factors that were associated with a lower risk of dying for both sexes and for both types of mitral valve replacement included atrial fibrillation and pacemaker implantation. 

The results of the sensitivity analysis are shown in Supplementary Table S3. After PSM, we found populations of men and women with very similar distributions of concomitant procedures such as coronary artery bypass graft, surgical aortic valve replacement, and other valve procedures on pulmonary or tricuspid valves. For our main outcome variables, the sensitivity analysis confirmed the results of the multivariable logistic regression showing a significantly higher IHM among men than women who underwent bioprosthetic mitral valve replacement.

Over time, the IHM decreased significantly regardless of the type of valve.

Finally, among patients who received bioprosthetic mitral valve, male sex was associated with a significantly higher IHM (OR 1.28; 95% CI 1.1–1.5), while no such association was found for mechanical mitral valve replacement (OR 1.05; 95% CI 0.97–1.14).

## 4. Discussion

Current guidelines recommend mitral valve repair over replacement for primary mitral valve disease [10]. Accordingly, the utilization of SMVR has steadily increased. Gammie et al. [18], in an eight-year review (2000–2007), reported that the rate of SMVR rose from 42 to 61% in the United States. Therefore, around 40% of patients with mitral valve disease need an SMVR. In Spain, we identified 44,340 hospitalizations of adults who underwent SMVR (2001–2015). Because of this, data surrounding the choice of prosthesis require further attention. 

The main finding of our investigation is the dramatic decrease in the number of patients who underwent a mechanical SMVR (91% in 2001 to 77% in 2015) while the use of bioprosthetic SMVR increased in both sexes. Bioprosthetic valves represented 9% of all valve replacements at the beginning of the study period and rose to 24% in 2015. Jamieson et al. [6] reported high rates of structural valve deterioration (SVD) of Carpentier-Edwards supra-annular porcine mitral bioprosthesis for patients younger than 70 years old but acceptable durability for patients older than 70 years. Considering this finding and documented clinical performance of other porcine valves, bioprosthetic valves are not recommended for non-repairable or failed repairs of the MV for patients less than 70 years old because of the limited durability of those valves [19,20]. 

The mean age of patients who underwent a bioprosthetic SMVR has significantly increased in Spain in both men and women. The age of patients undergoing a mechanical SMVR also increased significantly in both groups of patients (mean 61.2 years in men and 62.9 in women in 2001–2003 vs. 63.7 and 65.5 years old, respectively, in 2013–2015; all *p* < 0.001). These patients were younger than 70 years in accordance with the recommendations mentioned above. These trends have also been reported in other studies conducted in Europe and the U.S.A. for SAVR [21,22]. A substantial reduction in the rate of implanted aortic mechanical valves has also been noted in our country. At the same time, there has been an increase in the use of bioprosthetic valves [17], which allows the avoidance of permanent anticoagulation. 

Over the entire period, women were more likely to receive a mechanical or bioprosthetic SMVR. Women undergoing a SMVR were significantly older than men, consistent with results published by Seeburger et al. [11] in Germany and recently in a study performed to determine if there is a sex-based bias in treatment of mitral valve disease in the USA [23]. Kislitsina et al. [23] found that women tend to be referred for mitral valve surgery later in the course of the disease. In contrast with prior studies suggesting that women who undergo MV surgery have higher comorbidity rates than men [24], we found that the prevalence of heart failure was significantly higher among men than in women, and women had fewer comorbid conditions such as COPD, peripheral vascular disease, acute renal disease, coronary artery disease, endocarditis, renal disease, or liver disease. Logically, CCI was significantly lower for females. T2DM was more prevalent among women undergoing mechanical or bioprosthetic valve replacement. 

In our study, obesity was more prevalent among women than men for both mechanical and bioprosthetic valve replacements. A higher body mass index has been previously described as a positive predictor of outcomes after CABG with or without valve surgery [25]. Thus, the obesity-paradox might play a role in the improved outcomes observed in our study among women [26]. Obesity has also been found to be more prevalent in T2DM patients undergoing SAVR with a lower IHM [27]. These authors also found that obesity might contribute to this better outcome mentioned above.

The mortality rates for cardiac surgery have dramatically decreased [28], and as expected, we found that IHM of all types of SMVR has decreased significantly over the last 15 years regardless of sex, increasing age, and comorbidities. A similar trend was found for operative mortality in other studies concerning aortic valve surgery, which could reflect improvements in cardiac surgical technique over time and improved health care in general [29]. 

There are conflicting results in the literature regarding the influence of sex on in-hospital clinical outcomes in mitral or aortic valve surgery. Previous studies have found poorer outcomes of women compared to men undergoing surgery for mitral valve disease [30]. Wong et al. [12] found that female sex was associated with higher in-hospital MACCE (9.4% vs. 8.3% OR 1.33 95% CI 1.12–1.33). Female sex has also been described as a factor associated with IHM in aortic valve surgery in several studies [31]. However, men in our study who underwent a mechanical SMVR had significantly higher crude IHM (14 in 2001–2003 vs. 11 and 12 in 2013–2015 vs. 10% all *p* < 0.001) and higher rates of MACCE (19 in 2001–2003 vs. 13 and 17 in 2013–2015 vs. 12% *p* < 0.001) than women. After multivariate analysis of factors associated with IHM, it was noted that male sex was associated with increased mortality only in bioprosthetic mitral valve replacement (1.28 OR 95% CI 1.1–1.5). We suggest that the higher prevalence of comorbidities among men can mainly explain our results. In our population, many patients had undergone multiple interventions that could act as confounding factors. In order to control this, we used PSM and obtained the same results as the multivariable logistic regression. In contrast, one study has found that clinical outcomes are dependent upon the severity of the mitral disease and associated comorbidities at the time of surgery. Propensity score-matching of men and women with similar disease states clarified several of these issues and the apparent sex-based differences in outcomes disappeared [19]. Other studies have confirmed this observation reporting similar survival rates of mitral valve repair in men and women [32].

There are some points that should be taken into consideration when interpreting the results of the present study. This is a retrospective observational study using the SNHDD, an administrative database that contains discharge data for hospitalizations in Spain and uses information that the physician has included in the discharge report [14]. Coding practices, as well as errors in coding, may differ between individual physicians and institutions. In addition, in order to maintain confidentiality, the name of the hospitals is not provided by the Spanish National Hospital Discharge Database (SNHDD), and the number of procedures performed per year is not available. 

Our findings are limited by the lack of data on some relevant clinical and surgical aspects such as type of mitral valve disease, primary or secondary MR, information on valve structure, treatments during hospitalization, or left ventricular ejection fraction. Unfortunately, the SNHDD does not include any data on hemodynamic features. The absence of these parameters may affect the analysis and limit the generalizability of this study. We also lack information on surgery adjuvant techniques such as division of secondary chordae, placement of edge-to-edge stitches, and repositioning of papillary muscles, techniques that have been advocated to reduce the rate of recurrence [33]. Unfortunately, the SNHDD does not include details regarding the cause of death. Finally, in our investigation we analyzed the use of SMVR but not in those patients who underwent mitral valve plasty/repair that is currently a common procedure in many centers. Therefore, the influence of the use of valve repair in our results is not evaluated.

Despite these limitations, the quality and validity of our dataset have been assessed and determined to be useful for health research [34].

## 5. Conclusions

Our study reveals that the incidence of SMVR was higher in women, who were older than men at the time of surgery. Mechanical SMVR decreased and the use of bioprosthetic valves increased over time. IHM and MACCE decreased over time regardless of sex, age, and comorbidities. Women had better hospital outcomes in our study than men. For both sexes, higher mortality rates were associated with the presence of comorbidities and increasing age. Remarkably, after multivariable adjustment, IHM was higher among men who underwent bioprosthetic valves than women. However, given the methodological limitations of administrative data, more prospective investigations aimed at evaluating the influence of SMVR in patients with MV disease are needed.

## Figures and Tables

**Table 1:** Comorbid conditions in hospitalized patients undergoing mechanical mitral valve replacement in Spain from 2001 to 2015.

		2001–2003	2004–2006	2007–2009	2010–2012	2013–2015	Total	*p*-Value
Charlson Comorbidity Index, mean (SD) *	Men	0.90 (0.9)	1.00 (0.9)	1.03 (0.9)	1.07 (1.0)	1.15 (1.0)	1.03 (0.9)	<0.001
	Women	0.80 (0.9)	0.92 (0.9)	0.96 (0.9)	0.99 (0.9)	1.09 (1.0)	0.95 (0.9)	<0.001
Chronic obstructive pulmonary disease, *n* (%) *	Men	328 (11)	371 (12)	307 (11)	311 (11)	315 (11)	1632 (11)	0.304
	Women	149 (3)	173 (3)	139 (3)	176 (4)	157 (4)	794 (3)	0.063
Type 2 diabetes mellitus, *n* (%) *	Men	262 (9)	360 (12)	421(15)	441 (16)	521 (18)	2005 (14)	<0.001
	Women	623 (13)	807 (16)	785 (18)	765 (18)	785 (18)	3765 (16)	<0.001
Peripheral vascular disease, *n* (%) *	Men	95 (3)	158 (5)	175 (6)	184 (6)	196 (7)	808 (6)	<0.001
	Women	58 (1)	72 (1)	97 (2)	84 (2)	129 (3)	440 (2)	<0.001
Acute renal disease, *n* (%)	Men	264 (9)	309 (10)	366 (13)	447 (16)	563 (19)	1949 (13)	<0.001
	Women	261 (5)	315 (6)	388 (9)	487 (11)	534 (12)	1985 (9)	<0.001
Cerebrovascular disease, *n* (%) *	Men	123 (4)	131 (4)	117 (4)	121 (4)	186 (6)	678 (5)	<0.001
	Women	217 (4)	226 (5)	208 (5)	215 (5)	240 (5)	1106 (5)	0.145
Congestive heart failure, *n* (%) *	Men	610 (21)	671 (22)	633 (23)	655 (23)	810 (27)	3379 (23)	<0.001
	Women	907 (19)	953 (19)	884 (20)	821 (19)	1050 (24)	4615 (20)	<0.001
Atrial fibrillation, *n* (%) *	Men	1334 (47)	1502 (50)	1346 (48)	1385 (50)	1494 (51)	7061 (49)	0.026
	Women	2780 (58)	3148 (64)	2862 (64)	2714 (63)	2822 (65)	14,326 (63)	<0.001
Pulmonary hypertension, *n* (%) *	Men	576 (20)	630 (21)	578 (21)	564 (20)	626 (21)	2974 (21)	0.759
	Women	1351 (28)	1632 (33)	1463 (33)	1386 (32)	1469 (34)	7301 (32)	<0.001
Coronary artery disease, *n* (%) *	Men	749 (26)	820 (27)	747 (27)	811 (29)	899 (30)	4026 (28)	0.002
	Women	554 (12)	654 (13)	580 (13)	527 (12)	562 (13)	2877 (13)	0.111
Obesity, *n* (%) *	Men	86 (3)	110 (4)	112 (4)	174 (6)	209 (7)	691 (5)	<0.001
	Women	271 (6)	355 (7)	371 (8)	402 (9)	450 (10)	1849 (8)	<0.001
Cardiogenic shock, *n* (%) *	Men	136 (5)	135 (4)	139 (5)	169 (6)	172 (6)	751 (5)	0.033
	Women	135 (3)	151 (3)	149 (3)	149 (3)	111 (3)	695 (3)	0.082
Gastrointestinal hemorrhage, *n* (%)	Men	17 (1)	13 (0)	11 (0)	12 (0)	11 (0)	64 (0)	0.740
	Women	17 (0)	17 (0)	20 (0)	20 (0)	8 (0)	82 (0)	0.192
Endocarditis, *n* (%) *	Men	334 (12)	377 (13)	448 (16)	465 (16)	602 (20)	2226 (15)	<0.001
	Women	244 (5)	278 (6)	326 (7)	370 (9)	405 (9)	1623 (7)	<0.001
Pneumonia, *n* (%) *	Men	101 (3)	123 (4)	105 (4)	85 (3)	70 (2)	484 (3)	0.002
	Women	122 (3)	133 (3)	121 (3)	84 (2)	75 (2)	535 (2)	0.003
Renal disease, *n* (%) *	Men	158 (5)	213 (7)	204 (7)	255 (9)	272 (9)	1102 (8)	<0.001
	Women	133 (3)	203 (4)	216 (5)	247 (6)	324 (7)	1123 (5)	<0.001
Liver disease, *n* (%) *	Men	94 (3)	111 (4)	120 (4)	150 (5)	170 (6)	645 (4)	<0.001
	Women	95 (2)	129 (3)	130 (3)	153 (4)	170 (4)	677 (3)	<0.001
Cancer, *n* (%) *	Men	21 (1)	44 (1)	37 (1)	41 (1)	49 (2)	192 (1)	0.027
	Women	21 (0)	32 (1)	34 (1)	29 (1)	26 (1)	142 (1)	0.371
Weight loss, *n* (%)	Men	2 (0)	22 (1)	15 (0)	9 (0)	18 (1)	66 (0)	0.002
	Women	6 (0)	25 (0)	16 (0)	22 (0)	17 (0)	86 (0)	0.015

* *p* < 0.05 for difference when comparing men and women.

**Table 2:** Incidence, sociodemographic and clinical characteristics of hospitalized patients undergoing bioprosthetic mitral valve replacement in Spain from 2001 to 2015.

		2001–2003	2004–2006	2007–2009	2010–2012	2013–2015	Total	*p*-Value
Number of procedures (Incidence per 1,000,000 inhabitants)	Men	332 (7)	410 (8)	595 (11)	697 (12)	928 (17)	2962 (11)	<0.001
	Women	428 (8)	593 (11)	862 (15)	1065 (18)	1253 (21)	4201 (15)	<0.001
	Both	760 (7)	1003 (9)	1457 (13)	1762 (15)	2181 (19)	7163 (13)	<0.001
Age, mean (SD) *	Men	70.2 (11.0)	70.2 (10.4)	71.4 (10.4)	72.8 (8.8)	73.6 (7.9)	72.1 (9.5)	<0.001
	Women	71.0 (9.9)	72.5 (8.4)	72.7 (9.2)	74.0 (8.1)	74.6 (7.3)	73.4 (8.4)	<0.001
Rheumatic mitral insufficiency, *n* (%)	Men	15 (5)	18 (4)	21 (3)	22 (3)	27 (3)	102 (3)	0.732
	Women	16 (4)	24 (4)	32 (4)	30 (3)	33 (3)	180 (4)	0.580
Coronary artery bypass graft, *n* (%) *	Men	72 (22)	91 (22)	142 (24)	182 (26)	261 (28)	748 (25)	0.056
	Women	63 (15)	83 (14)	113 (13)	142 (13)	148 (12)	549 (13)	0.511
Surgical aortic valve replacement, *n* (%) *	Men	87 (26)	146 (36)	227 (38)	266 (38)	343 (37)	1069 (36)	0.002
	Women	125 (29)	188 (32)	274 (32)	362 (34)	445 (35)	1394 (33)	0.100
Other valve procedures: pulmonary or tricuspid valves, *n* (%) *	Men	41 (12)	58 (14)	110 (19)	140 (20)	213 (23)	562 (19)	<0.001
	Women	90 (21)	169 (28)	287 (33)	376 (35)	449 (36)	1371 (33)	<0.001
Intra-aortic balloon counterpulsation, *n* (%) *	Men	23 (7)	31 (8)	48 (8)	54 (8)	60 (6)	216 (7)	0.774
	Women	16 (4)	20 (3)	39 (4)	45 (4)	60 (5)	180 (4)	0.659
Pacemaker implantation, *n* (%) *	Men	23 (7)	28 (7)	47 (8)	49 (7)	82 (9)	229 (8)	0.581
	Women	19 (4)	29 (5)	45 (5)	66 (6)	77 (6)	236 (6)	0.509
Blood transfusion, *n* (%)	Men	67 (20)	97 (24)	140 (23)	195 (28)	288 (31)	787 (27)	<0.001
	Women	100 (23)	150 (25)	198 (23)	303 (28)	367 (29)	1118 (27)	0.004
Length of hospital stay, mean (SD) *	Men	29.0 (25)	27.3 (24)	26.7 (25)	26.4 (25)	24.2 (24)	26.2 (25)	0.019
	Women	25.3 (22)	25.7 (25)	25.0 (26)	22.7 (22)	20.5 (17)	23.2 (22)	<0.001
In-hospital mortality, *n* (%) *	Men	61 (18)	59 (14)	118 (20)	104 (15)	129 (14)	471 (16)	0.015
	Women	79 (18)	84 (14)	123 (14)	154 (14)	148 (12)	588 (14)	0.015
MACCE, *n* (%) *	Men	88 (26)	83 (20)	151 (25)	138 (20)	168 (18)	628 (21)	0.001
	Women	93 (22)	110 (18)	154 (18)	195 (18)	192 (15)	744 (18)	0.037

MACCE: in-hospital all-cause death, acute myocardial infarction or ischemic stroke. * *p* < 0.05 for difference when comparing men and women.

**Table 3:** Comorbidity of patients hospitalized that underwent a bioprosthetic mitral valve replacement in Spain from 2001 to 2015.

		2001–2003	2004–2006	2007–2009	2010–2012	2013–202015	Total	*p*-Value
Charlson Comorbidity Index, mean (SD) *	Men	1.11 (1.1)	1.17 (1.0)	1.24 (1.0)	1.24 (1.0)	1.37 (1.1)	1.26 (1.0)	<0.001
	Women	0.93 (0.9)	1.04 (0.9)	1.14 (1.0)	1.10 (1.0)	1.23 (1.0)	1.12 (1.0)	<0.001
Chronic obstructive pulmonary disease, *n* (%)	Men	47 (14)	61 (15)	81 (14)	89 (13)	143 (15)	421 (14)	0.626
	Women	10 (2)	18 (3)	26 (3)	35 (3)	38 (3)	127 (3)	0.919
Type 2 diabetes mellitus, *n* (%) *	Men	52 (16)	64 (16)	102 (17)	116 (17)	172 (18)	506 (17)	0.631
	Women	69 (16)	99 (17)	174 (20)	202 (19)	247 (18)	791 (19)	0.239
Peripheral vascular disease, *n* (%) *	Men	18 (5)	24 (6)	37 (6)	69 (10)	69 (7)	217 (7)	0.027
	Women	9 (2)	13 (2)	33 (4)	39 (4)	35 (3)	129 (3)	0.190
Acute renal disease, *n* (%)	Men	54 (16)	81 (20)	116 (19)	170 (24)	242 (26)	663 (22)	<0.001
	Women	41 (10)	62 (10)	124 (15)	164 (15)	233 (19)	624 (15)	<0.001
Cerebrovascular disease, *n* (%)	Men	14 (4)	22 (5)	22 (4)	46 (7)	45 (5)	149 (5)	0.170
	Women	17 (4)	30 (5)	36 (4)	63 (6)	75 (6)	221 (5)	0.219
Congestive heart failure, *n* (%) *	Men	81 (24)	107 (26)	170 (29)	196 (28)	310 (33)	864 (29)	0.007
	Women	110 (26)	157 (26)	222 (26)	270 (25)	383 (31)	1142 (27)	0.032
Atrial fibrillation, *n* (%) *	Men	123 (37)	176 (43)	283 (48)	332 (48)	474 (51)	1388 (47)	<0.001
	Women	217 (51)	379 (64)	529 (61)	655 (61)	829 (66)	2609 (62)	<0.001
Pulmonary hypertension, *n* (%) *	Men	52 (16)	87 (21)	138 (23)	148 (21)	238 (26)	663 (22)	0.004
	Women	104 (24)	200 (34)	317 (37)	329 (31)	431 (34)	1381 (33)	<0.001
Coronary artery disease, *n* (%) *	Men	109 (33)	151 (37)	218 (37)	249 (36)	365 (39)	1092 (37)	0.272
	Women	92 (21)	139 (23)	176 (20)	248 (23)	273 (22)	928 (22)	0.546
Obesity, *n* (%) *	Men	7 (2)	11 (3)	23 (4)	38 (5)	54 (6)	133 (4)	0.010
	Women	29 (7)	34 (6)	59 (7)	75 (7)	114 (9)	311 (7)	0.075
Cardiogenic shock, *n* (%) *	Men	23 (7)	29 (7)	39 (6)	47 (7)	54 (6)	192 (6)	0.894
	Women	24 (6)	24 (4)	47 (5)	44 (4)	47 (4)	186 (4)	0.255
Gastrointestinal hemorrhage, *n* (%)	Men	1 (0)	4 (1)	2 (0)	7 (1)	3 (0)	17 (1)	0.243
	Women	10 (2)	5 (1)	3 (0.3)	4 (0)	6 (0)	28 (1)	<0.001
Endocarditis, *n* (%) *	Men	55 (17)	87 (21)	133 (22)	183 (26)	260 (28)	718 (24)	<0.001
	Women	47 (11)	59 (10)	112 (13)	188 (18)	240 (19)	646 (15)	<0.001
Pneumonia, *n* (%) *	Men	18 (5)	17 (4)	28 (5)	27 (4)	50 (5)	140 (5)	0.611
	Women	23 (5)	24 (4)	21 (2)	26 (2)	33 (3)	127 (3)	0.011
Renal disease, *n* (%) *	Men	24 (7)	49 (12)	71 (12)	87 (12)	134 (14)	365 (12)	0.018
	Women	16 (4)	36 (6)	58 (7)	85 (8)	141 (11)	336 (8)	<0.001
Liver disease, *n* (%) *	Men	15 (4)	15 (4)	24 (4)	36 (5)	55 (6)	145 (5)	0.328
	Women	10 (2)	16 (3)	27 (3)	30 (3)	45 (4)	128 (3)	0.655
Cancer, *n* (%) *	Men	6 (2)	6 (1)	9 (1)	16 (2)	19 (2)	56 (2)	0.806
	Women	5 (1)	2 (0)	10 (1)	9 (1)	7 (1)	33 (1)	0.313
Weight loss, *n* (%)	Men	1 (0)	0 (0)	2 (0)	3 (0)	4 (0)	10 (0)	0.769
	Women	0 (0)	2 (0)	2 (0)	5 (0)	7 (1)	16 (0)	0.488

* *p* < 0.05 for difference when comparing men and women.

**Table 4:** Multivariable analysis of factors associated with in-hospital mortality among women and men undergoing mitral valve replacement according to the type of valve.

	Mechanical Mitral Valve Replacement			Bioprosthetic Mitral Valve Replacement		
	Men	Women	Both	Men	Women	Both
	OR (95% CI)	OR (95% CI)	OR (95% CI)	OR (95% CI)	OR (95% CI)	OR (95% CI)
Age	1.06 (1.05–1.06)	1.05 (1.05–1.06)	1.05 (1.05–1.06)	1.03 (1.02–1.05)	1.03 (1.01–1.04)	1.03 (1.02–1.04)
Surgical aortic valve replacement		1.24 (1.11–1.38)	1.17 (1.08–1.27)	1.32 (1.05–1.67)	1.22 (0.99–1.52)	1.26 (1.08–1.48)
Other valve procedures: pulmonary or tricuspid valves	1.2 (1.03–1.4)	1.21 (1.08–1.35)	1.2 (1.1–1.32)			
Intra-aortic balloon counterpulsation	2.93 (2.4–3.59)	3.39 (2.71–4.23)	3.07 (2.65–3.57)	3.28 (2.32–4.64)	5.64 (3.88–8.19)	4.09 (3.18–5.27)
Pacemaker implantation	0.62 (0.46–0.83)	0.57 (0.43–0.76)	0.6 (0.49–0.73)	0.53 (0.32–0.87)	0.31 (0.16–0.59)	0.44 (0.3–0.64)
Blood transfusion	1.56 (1.38–1.76)	1.49 (1.34–1.66)	1.52 (1.4–1.65)		1.66 (1.34–2.05)	1.35 (1.15–1.58)
Peripheral vascular disease					2.42 (1.49–3.93)	1.56 (1.14–2.13)
Acute Renal disease	3.58 (3.13–4.08)	4.25 (3.75–4.83)	3.92 (3.57–4.29)	2.54 (2–3.21)	3.39 (2.68–4.28)	2.83 (2.4–3.35)
Cerebrovascular disease	1.67 (1.33–2.11)	1.75 (1.44–2.13)	1.71 (1.47–1.99)		1.83 (1.24–2.7)	1.61 (1.21–2.16)
Congestive heart failure	1.34 (1.18–1.52)	1.82 (1.63–2.04)	1.59 (1.47–1.73)	1.68 (1.34–2.11)	1.55 (1.25–1.92)	1.64 (1.4–1.91)
Atrial fibrillation	0.56 (0.5–0.63)	0.48 (0.44–0.54)	0.51 (0.47–0.56)	0.59 (0.47–0.75)	0.63 (0.51–0.78)	0.6 (0.52–0.7)
Coronary artery disease	1.42 (1.21–1.66)	1.49 (1.31–1.7)	1.48 (1.32–1.66)			
Cardiogenic shock	5.09 (4.22–6.15)	8.17 (6.75–9.89)	6.38 (5.58–7.29)	4.5 (3.15–6.43)	8.83 (6.04–12.91)	6.09 (4.72–7.87)
Gastrointestinal hemorrhage	2.06 (1.13–3.76)	3.34 (2–5.58)	2.74 (1.86–4.05)			
Endocarditis	2.14 (1.86–2.47)	1.66 (1.41–1.94)	1.89 (1.7–2.1)	1.44 (1.12–1.86)	1.6 (1.24–2.07)	1.5 (1.25–1.79)
Pneumonia	2.53 (2.01–3.18)	3.15 (2.53–3.93)	2.85 (2.43–3.34)	2.3 (1.52–3.48)	4.02 (2.61–6.19)	2.95 (2.19–3.98)
Renal disease	1.66 (1.39–1.98)	1.79 (1.5–2.14)	1.73 (1.53–1.96)		1.97 (1.45–2.69)	1.59 (1.28–1.98)
Liver disease	3.62 (2.93–4.47)	4.33 (3.53–5.32)	3.93 (3.4–4.55)	3.06 (2.04–4.59)	3.99 (2.55–6.26)	3.51 (2.6–4.73)
Cancer	1.6 (1.06–2.42)		1.47 (1.06–2.04)			
Year	0.92 (0.90–0.93)	0.92 (0.91–0.93)	0.92 (0.91–0.93)	0.94 (0.92–0.97)	0.93 (0.9–0.95)	0.93 (0.92–0.95)
Male sex			1.05 (0.97–1.14)			1.28 (1.1–1.5)

## Supplementary Materials

The following are available online at https://www.mdpi.com/2077-0383/9/12/4108/s1, Table S1: ICD-9-CM codes for the clinical diagnosis and procedures used in this investigation, Table S2: Incidence, sociodemographic and clinical characteristics of hospitalized patients undergoing mechanical mitral valve replacement in Spain from 2001 to 2015, Table S3: Distribution according to study variables and hospital outcomes of propensity score–matched men and women who underwent a mechanical or bioprosthetic surgical mitral valve replacement.
