# Supplementary material for: The Influence of Sex on Clinical Outcomes after Surgical Mitral Valve Replacement in Spain (2001–2015)
Source: J Clin Med. 2020 Dec 19;9(12):4108. doi: 10.3390/jcm9124108 (PMC7766549; doi:10.3390/jcm9124108)
Supplement: Supplementary file 1 [file jcm-09-04108-s001.pdf]

Table S1. ICD-9-CM codes for the clinical diagnosis and procedures used in this investigation.

	ICD-9-CM Codes
Chronic obstructive pulmonary disease	490, 491, 491.0, 491.1, 491.2x, 491.8, 491.9, 492, 492.0, 492.8, 496
Type 2 diabetes mellitus	250.x0 and 250.x2
Peripheral vascular disease	0.93.0, 473.3, 440.x, 441.x, 443.1–443.9, 447.1, 557.1, 557.9, V43.4
Renal disease	403.01, 403.11, 403.91, 404.02, 404.03, 404.12, 404.13, 404.92, 404.93, 582, 583.0–583.7, 585, 586, 588.0, V42.0, V45.1, V56
Cerebrovascular disease	362.34, 430.x–438.x
Congestive heart failure	398.91, 402.01, 402.11, 402.91, 404.01, 404.03, 404.11, 404.13, 404.91, 404.93, 425.4–425.9, 428.x
Ischemic stroke	433.xx, 434,xx, 436
Atrial fibrillation	427.31
Pulmonary hypertension	416.0 and 416.8
Coronary artery disease	410.xx, 412.x, 413.x, 414.0, 414, 414.00, 414.01, 414.2-9
Acute myocardial infarction	410.xx
Obesity	278.0
Cardiogenic shock	785.51
Gastrointestinal hemorrhage	578, 578.0, 578.1, 578.9
Endocarditis	424.90, 421.9, 424.1, 421.0
Pneumonia	480–488, 507.0–507.8
Acute renal disease	584, 584.5–584.9
Liver disease	070.22, 070.23, 070.32, 070.33, 070.44, 070.54, 070.6, 070.9, 456.0–456.2, 570.x, 571.x, 572.2–572.8, 573.3, 573.4, 573.8, 573.9, V42.7
Cancer	140.x–172.x, 174.x–195.x, 196.x–199.x
Weight loss	260, 261, 262, 263.0–263.2, 263.8, 263.9, 783.2, 977.4
Coronary artery bypass graft	36.10–36.19
Surgical aortic valve replacement	35.21, 35.22
Other valve procedures: pulmonary or tricuspid valves	35.33, 35.03, 35.04, 35.13, 35.14, 35.25, 35.26, 35.27, 35.28, 35.33
Intra-aortic balloon counterpulsation	37.61
Pacemaker implantation	37.70–37.74; 37.80–37.83
Blood transfusion	99.00, 99.01–99.08

Table S2. Incidence, sociodemographic and clinical characteristics of hospitalized patients undergoing mechanical mitral valve replacement in Spain from 2001 to 2015.

		2001–2003	2004–2006	2007–2009	2010–2012	2013–2015	Total	*p*-Value
Number of procedures (Incidence per 1,000,000 inhabitants)	Men	2857 (57)	3002 (56)	2776 (50)	2811 (49)	2948 (52)	14,394 (53)	<0.001
	Women	4771 (90)	4941 (89)	4460 (77)	4278 (72)	4333 (73)	22,783 (80)	<0.001
	Both	7628 (74)	7943 (73)	7236 (63)	7089 (61)	7281 (63)	37,177 (67)	<0.001
Age, mean (SD) *	Men	61.2 (11)	62.0 (11)	62.0 (12)	63.4 (13)	63.7 (11)	62.5 (11)	<0.001
	Women	62.9 (10)	64.0 (10)	64.4 (10)	64.9 (11)	65.5 (10)	64.3 (10)	<0.001
Rheumatic mitral insufficiency, *n* (%)	Men	97 (3)	108 (4)	115 (4)	123 (4)	122 (4)	565 (4)	0.675
	Women	179 (4)	182 (4)	186 (4)	178 (4)	192 (4)	917 (4)	0.453
Coronary artery bypass graft, *n* (%) *	Men	475 (17)	522 (17)	457 (17)	513 (18)	544 (18)	2511 (17)	0.162
	Women	284 (6)	350 (7)	319 (7)	282 (7)	281 (6)	1516 (7)	0.123

Surgical aortic valve replacement, *n* (%) *	Men	898 (31)	1004 (33)	960 (35)	991 (35)	1072 (36)	4925 (34)	<0.001
	Women	1367 (29)	1537 (31)	1410 (32)	1383 (32)	1418 (33)	7115 (31)	<0.001
Other valve procedures: pulmonary or tricuspid valves, *n* (%) *	Men	367 (13)	465 (15)	498 (18)	517 (18)	581 (20)	2428 (17)	<0.001
	Women	1174 (25)	1435 (20)	1422 (32)	1445 (34)	1566 (36)	7042 (31)	<0.001
Intra-aortic balloon counterpulsation, *n* (%) *	Men	124 (4)	149 (5)	147 (5)	162 (6)	151 (5)	733 (5)	0.177
	Women	99 (2)	120 (2)	115 (3)	125 (3)	99 (2)	558 (2)	0.107
Pacemaker implantation, *n* (%)	Men	91 (3)	123 (4)	102 (4)	147 (5)	169 (6)	632 (4)	<0.001
	Women	144 (3)	149 (3)	200 (4)	223 (5)	225 (5)	941 (4)	<0.001
Blood transfusion, *n* (%)	Men	684 (24)	685 (23)	618 (22)	698 (25)	758 (26)	3443 (24)	0.013
	Women	1114 (23)	1210 (24)	1028 (23)	991 (23)	1127 (26)	5470 (24)	0.004
Length of hospital stay, mean (SD)*	Men	24.07 (21)	24.14 (22)	24.49 (24)	23.11 (23)	21.14 (20)	23.38 (22)	<0.001
	Women	22.33 (18)	21.84 (20)	21.86 (19)	21.30 (22)	18.60 (18)	21.23 (19)	<0.001
In-hospital mortality, *n* (%) *	Men	407 (14)	396 (13)	339 (12)	339 (12)	300 (10)	1781 (12)	<0.001
	Women	527 (11)	503 (10)	423 (10)	432 (10)	304 (7)	2189 (10)	<0.001
MACCE, *n* (%) *	Men	535 (19)	549 (18)	478 (17)	471 (17)	458 (15)	2491 (17)	0.011
	Women	628 (13)	632 (13)	531 (12)	555 (13)	413 (9)	2759 (12)	<0.001

Table S3. Distribution according to study variables and hospital outcomes of propensity score–matched men and women who underwent a mechanical or bioprosthetic surgical mitral valve replacement.

		**Mechanical**			**Bioprosthetic**		
		**Men**	**Women**	***p***	**Men**	**Women**	***p***
Age, mean (SD)		62.5 (11)	62.5 (11)	0.767	72.1 (9)	72.6 (9)	0.072
Coronary artery bypass graft. *n* (%)	No	11,883 (83)	11,838 (82)	0.169	2214 (75)	2420 (82)	<0.001
	Yes	2511 (17)	2601 (18)		748 (25)	542 (18)	
Surgical aortic valve replacement. *n* (%)	No	9469 (66)	9518 (66)	0.593	1893 (64)	1914 (65)	0.569
	Yes	4925 (34)	4876 (33)		1069 (36)	1048 (35)	
Other valve procedures: pulmonary or tricuspid valves. *n* (%)	No	13,526 (94)	13,456 (93)	0.204	2784 (94)	2763 (93)	0263
	Yes	868 (6)	938 (6)		178 (6)	199 (7)	
Intra-aortic balloon counterpulsation. *n* (%)	No	13,661 (95)	13,910 (96)	<0.001	2746 (93)	2797 (94)	0.007
	Yes	733 (5)	484 (3)		216 (7)	165 (6)	
Pacemaker implantation. *n* (%)	No	13,762 (96)	13,790 (95)	0.416	2733 (92)	2766 (93)	0.097
	Yes	632 (4)	604 (4)		229 (8)	196 (7)	
Charlson Comorbidity Index. mean (SD)		1.03 (1.02)	0.89 (1.03)	<0.001	1.26 (1.14)	1.1 (112)	<0.001
Chronic obstructive pulmonary disease. *n* (%)	No	12,762 (87)	13,606 (94)	<0.001	2541 (86)	2835 (967)	<0.001
	Yes	1632 (11)	788 (5)		421 (14)	127 (4)	

Type 2 diabetes mellitus. *n* (%)	No	12,389 (86)	12,550 (87)	0.013	2456 (83)	2469 (83)	0.652
	Yes	2005 (14)	1844 (12)		506 (17)	493 (17)	
Peripheral vascular disease. *n* (%)	No	13,586 (95)	13,968 (97)	<0.001	2745 (93)	2835 (96)	<0.001
	Yes	808 (6)	426 (3)		217 (7)	127 (4)	
Cerebrovascular disease. *n* (%)	No	13,716 (95)	13,768 (95)	0.126	2813 (95)	2814 (95)	0.955
	Yes	678 (5)	626 (4)		149 (5)	148 (5)	
Congestive heart failure. *n* (%)	No	11,015 (76)	11,373 (79)	<0.001	2098 (71)	2134 (72)	0.301
	Yes	3379 (23)	3021 (21)		864 (29)	828 (28)	
Atrial fibrillation. *n* (%)	No	7333 (51)	6764 (47)	<0.001	1574 (53)	1440 (49)	<0.001
	Yes	7061 (49)	7630 (53)		1388 (47)	1522 (51)	
Pulmonary hypertension. *n* (%)	No	11,420 (80)	11,273 (78)	0.016	2299 (78)	2239 (76)	0.066
	Yes	2974 (21)	3121 (21)		663 (22)	723 (24)	
Coronary artery disease. *n* (%)	No	10,368 (72)	11,678 (81)	<0.001	1870 (63)	2093 (71)	<0.001
	Yes	4026 (28)	2716 (19)		1092 (37)	869 (29)	
Obesity. *n* (%)	No	13,703 (95)	13,726 (95)	0.661	2829 (96)	2829 (95)	0.999
	Yes	691 (5)	668 (4)		133 (4)	133 (4)	
Cardiogenic shock. *n* (%)	No	13,643 (95)	13,842 (96)	<0.001	2770 (93)	2797 (94)	0.141
	Yes	751 (5)	552 (4)		192 (6)	165 (6)	
Endocarditis. *n* (%)	No	12,168 (84)	12,885 (89)	<0.001	2244 (76)	2360 (80)	<0.001
	Yes	2226 (15)	1509 (10)		718 (24)	602 (20)	
Pneumonia. *n* (%)	No	13,910 (97)	14,008 (97)	0.001	2822 (95)	2843 (90)	0.182
	Yes	484 (3)	386 (3)		140 (5)	119 (4)	
Renal disease. *n* (%)	No	13,292 (92)	13,509 (94)	<0.001	2597 (88)	2666 (90)	0.004
	Yes	1102 (8)	885 (6)		365 (12)	296 (10)	
Liver disease. *n* (%)	No	13,749 (95)	13,861 (96)	0.002	2817 (95)	2850 (96)	0.035
	Yes	645 (4)	533 (4)		145(5)	112 (4)	
Cancer. *n* (%)	No	14,202 (99)	14,260 (99)	0.001	2906 (98)	2929 (99)	0.014
	Yes	192 (1)	134 (2)		56 (2)	33 (1)	
Length of hospital stay. mean (SD)		23.38 (22)	21.78 (21)	<0.001	26.19 (25)	24.32 (24)	0.003
In-hospital mortality. *n* (%)	No	12,613 (88)	12,683 (88)	0.206	2491 (84)	2557 (86)	0.015
	Yes	1781 (12)	1711 (12)		471 (16)	405 (14)	
